# Application of elastography to diagnose adenomyosis and evaluate the degree of dysmenorrhea: a prospective observational study

**DOI:** 10.1186/s12958-023-01145-y

**Published:** 2023-10-26

**Authors:** Qianhui Ren, Xiangyi Dong, Ming Yuan, Xue Jiao, Hao Sun, Zangyu Pan, Xinyu Wang, Guowei Tao, Wang Guoyun

**Affiliations:** 1 Department of Obstetrics and Gynecology, Shandong Provincial Hospital, Shandong University, No. 324 Jingwu Road, Jinan, 250021 Shandong China; 2 Cheeloo College of Medicine, Shandong University, Jinan, China; 3 Department of Ultrasonic Medicine, Qilu Hospital of Shandong University, No. 107 Wenhuaxi Road, Jinan, 250012 China; 4 JiNan Key Laboratory of Diagnosis and Treatment of Major Gynaecological Disease, Jinan, Shandong Province China; 5 Gynecology Laboratory, Shandong Provincial Hospital, Jinan, Shandong Province China; 6 Gynecology Laboratory, Medical Science and Technology Innovation Center, Shandong First Medical University & Shandong Academy of Medical Sciences, Jinan, Shandong Province, China

**Keywords:** Adenomyosis, Diagnosis, Dysmenorrhea, Elastography, Fibrosis

## Abstract

**Background:**

To determine whether there is a correlation between stiffness measured by strain elastography and the severity of dysmenorrhea and to determine the value of elastography in evaluating severe dysmenorrhea in patients with adenomyosis.

**Methods:**

The correlation between tissue stiffness and dysmenorrhea was analyzed by performing elastography on premenopausal women diagnosed with adenomyosis. Expression levels of transforming growth factor-β (TGF-β), α-smooth muscle actin (α-SMA), and protein gene product 9.5 (PGP9.5) were detected by immunohistochemistry; the correlation of TGF-β and α-SMA levels with the tissue stiffness and the degree of fibrosis was further analyzed. Also, the relationship of the PGP9.5 expression level with the tissue stiffness and degree of dysmenorrhea was determined.

**Results:**

The degree of dysmenorrhea was significantly positively correlated with lesion stiffness in patients with adenomyosis but not with the uterine or lesion volume. The cutoff for the strain ratio was > 1.36 between the adenomyosis and control groups, with an area under the curve (AUC) of 0.987. For severe dysmenorrhea, the cutoff for the strain ratio was > 1.65 in patients with adenomyosis, with an AUC of 0.849. TGF-β, α-SMA, and PGP9.5 expression levels were higher in adenomyotic lesions than in the endometrium of the adenomyosis and control groups. Both TGF-β and α-SMA levels were positively correlated with the tissue stiffness and degree of fibrosis. Additionally, the expression level of PGP9.5 showed a positive correlation with the tissue stiffness and degree of dysmenorrhea.

**Conclusions:**

Elastography can be used to evaluate the degree of dysmenorrhea; the greater the tissue stiffness, the greater the degree of dysmenorrhea. In addition, elastography performed well in the diagnosis of adenomyosis and the evaluation of severe dysmenorrhea in patients with adenomyosis.

**Supplementary Information:**

The online version contains supplementary material available at 10.1186/s12958-023-01145-y.

## Background

Adenomyosis is a common benign gynaecological condition characterized by ectopic endometrial glands and stroma within the myometrium. The prevalence of adenomyosis ranges widely, from 5 to 70%, in women who undergo hysterectomies. This variation in rates may be due to various diagnostic criteria, different patient populations, and pathologist bias [1, 2]. Adenomyosis induces progressive dysmenorrhea, menorrhagia, and infertility, adversely affecting women’s quality of life. Adenomyosis was also associated with a more than doubled risk of miscarriage [3]; however, it is important to note that up to 35% of women show no symptoms [3], which can easily lead to delayed diagnosis. Consequently, it is necessary to find a possible approach to diagnose adenomyosis early and evaluate its progress.

Emerging evidence has shown that repeated tissue injury and repair (ReTIAR) play an important role in the development of adenomyosis [4–6], eventually leading to fibrogenesis through an epithelial-mesenchymal transition (EMT) and fibroblast-to-myofibroblast transition (FMT), similar to endometriosis. Hence, the extent of lesion fibrosis may reflect the severity of adenomyosis.

Transvaginal ultrasound (TVUS) is a common first-line imaging diagnostic method for adenomyosis with good specificity and sensitivity, similar to those of magnetic resonance imaging (MRI) [6–9]. Compared with MRI, TVUS has the advantage of being repeatable, less costly, and widely available [3, 7, 9]. However, sonographers still face challenges when interpreting the ultrasound features associated with adenomyosis, most of which depend on subjective pattern recognition rather than objective measurement parameters [10]. Elastography, as an emerging imaging technique, can objectively reflect tissue stiffness. A positive correlation between tissue stiffness assessed by elastography and the degree of fibrosis has been reported [11]. Elastography could be mainly divided into two categories: stain imaging and shear-wave imaging. The tissue response to mechanical stimuli is used to quantify the tissue stiffness in both approaches [9, 11]. Elastography has proven useful in assessing the extent of tissue fibrosis and diagnosing benign and malignant diseases in the liver, breast, and other organs [12]. However, studies of elastography in gynaecology have only recently begun. Lesion stiffness in both adenomyosis and deep infiltrating endometriosis is stiffer than in adjacent normal tissues. It is reported that elastography may be promising in diagnosing and characterizing pelvic endometriotic lesions and adenomyosis [11].

This study aimed to determine whether a correlation exists between stiffness measured by strain elastography and the degree of dysmenorrhea. Further, Masson’s trichrome staining and immunohistochemistry (IHC) were performed to determine the extent of fibrosis and expression levels of transforming growth factor-β (TGF-β); α-smooth muscle actin (α-SMA), a marker of fibrosis; and protein gene product 9.5 (PGP9.5), a marker of nerve fibres, in order to evaluate the relationship of these markers with lesion stiffness and the degree of dysmenorrhea.

## Methods

### Patients and specimens

We enrolled patients who visited Qilu Hospital, Shandong University, between September 2021 and October 2022. The inclusion criteria for the study group were as follows: 1) patients diagnosed with adenomyosis based on clinical symptoms and imaging findings such as ultrasound, 2) premenopausal patients aged 18–50 years, 3) patients with dysmenorrhea with or without menorrhagia, 4) patients who had not received hormone therapy in the last three months, and 5) patients without reproductive system malignancies or pelvic inflammatory disease. Premenopausal patients without myometrial lesions or a history of reproductive system malignancies or infection were included in the control group. Further, non-sexual or menopausal women, patients with a previous or current history of reproductive system malignancies, and pregnant patients or those with coinfection of the reproductive system were excluded from the study. Finally, we recruited 39 premenopausal women as the control group and 57 premenopausal women as the adenomyosis group.

Demographic data and detailed medical histories were recorded before TVUS. Demographic information, including age, body mass index (BMI; kg/m^2^), age at menarche, gravidity, parity, mode of delivery, frequency and duration of menstrual periods, amount of menses, degree of dysmenorrhea, medication, and surgical history, were collected and recorded using questionnaires. All patients’ medical records, including intraoperative and pathological findings, were also recorded. The degree of dysmenorrhea was quantified using a numerical rating scale (NRS) from 0–10, with 0 representing no pain and 10 representing maximum pain. NRS scores were grouped into three levels: 0–3, none or mild; 4–6, moderate; and 7–10, severe. The amount of menses was recorded as mild, moderate, or severe based on the subjective evaluation of the patients.

The eutopic endometrium and adenomyotic tissue samples were collected from 20 patients with adenomyosis who underwent surgery. For the control group, specimens of the eutopic endometrial and normal myometrial tissues were obtained from 15 patients who underwent hysterectomy.

This study was approved by the ethics committee of the Medical Integration and Practice Center, Shandong University (approval number SDULCLL2022-1–21). All patients included in the study signed informed consent forms.

### Evaluation of the conventional TVUS and Elastography

TVUS and strain elastography were performed by a single-trained ultrasound specialist with several years of experience in gynaecological sonography, especially for adenomyosis. All enrolled patients underwent both conventional TVUS and strain elastography using the Nuewa R9 with a DE10-3WU transvaginal probe (Mindray, Shenzhen, China) less than 1 d preoperatively or during the outpatient examination. The sonographer was blinded to patients’ clinical information. Conventional TVUS was initially performed to diagnose adenomyosis according to the Morphological Uterus Sonographic Assessment criteria [13, 14]. The following information was recorded: volume of the uterus, thickness of the anterior and posterior walls, site and extent of typical adenomyotic lesions, and presence of ovarian endomyoma, endometrial polyps, and uterine fibroids. The volume of the uterus was recorded in the control group. The uterine and lesion volumes were calculated using the following formula for an ovoid: volume = D1 × D2 × D3 × 0.52, where D1, D2, and D3 represent the vertical, transverse, and anteroposterior diameters of the uterus or lesion, respectively.

Strain elastography was subsequently performed on all recruited patients. The elastograms of typical lesions were visualized in real time, followed by a B-mode image. To evaluate the strain ratio of the lesion, we applied external pressure using an ultrasound probe to produce a deformation. Three cycles of gentle compression and decompression were then performed. The elastogram images were colour-coded as red, yellow, green, and blue. The different colours represented the tissue stiffness relative to that of the endometrium or adjacent bowel. Blue represented the softest tissue, and red represented the hardest tissue. For the adenomyosis group, a region of interest (ROI) was set in the typical lesion area of the uterus, while another region of interest (Ref) was set in the adjacent normal myometrium. The stiffness of the lesion was semi-quantified using the ratio of Ref/ROI. The higher the ratio, the greater the stiffness of the lesion. For the control group, we set two regions of interest (ROI1 and ROI2) in the uterus, and the ratio of ROI1/ROI2 represented the stiffness of the normal myometrium.

To eliminate possible bias and maintain consistency, three strain ratios were measured for each patient, and the mean value was used as the stiffness of the lesion or normal myometrium.

### IHC Analysis

Serial 4-mm sections were obtained from each block. Routine deparaffinization and rehydration were performed. The first resultant slide was stained with hematoxylin and eosin (H&E) to confirm the pathological diagnosis.

Then, adenomyotic lesions and the eutopic endometrium of patients with adenomyosis and the control group underwent IHC staining for TGF-β, α-SMA, and PGP9.5. For antigen retrieval, the EDTA buffer (pH = 8.0) was microwaved on high for 5 min to a boil, and then the sections were heated in EDTA buffer on low for 15 min and cooled to room temperature. Next, the sections were treated with 3% hydrogen peroxide to block the activity of endogenous peroxidase for 30 min at 37℃. After the samples were blocked with 5% bovine serum albumin (BSA; Boster, Wuhan, China) for 50 min at 37℃, they were incubated with the primary antibody against TGF-β (1:100; Abcam, Cambridge, England), α-SMA (1:100; Abcam), and PGP9.5 (1:250; Abcam) overnight at 4℃. The sections were rinsed three times with PBS buffer and incubated with the horseradish peroxidase (HRP)-labelled secondary anti-rabbit/mouse antibody for 30 min at 37℃. The sections were then washed with PBS and treated with glucose oxidase-diaminobenzidine for microscopic observation. Finally, they were incubated with hematoxylin for 30 s, differentiated, and stained with anti-blue. The expression level of the targeted substance was semi-quantitatively calculated based on the proportion of positive cell areas using ImageJ software (National Institutes of Health, USA). To reduce bias and ensure reliability, a series of 3–5 randomly selected images for every section were taken to obtain a mean value.

### Masson’s Trichrome staining

Masson’s trichrome staining was used to detect collagen fibres in the lesion tissue in adenomyosis samples and the normal myometrium in control samples; it was performed according to common protocols using a Masson’s trichrome staining kit (Solarbio, Beijing, China). After Masson’s trichrome staining, images were observed and captured using a microscope. Collagen fibres appeared blue, whereas smooth muscle fibres and red blood cells appeared red. The extent of fibrosis was represented by the relative content of collagen fibres, which was evaluated by the ratio of positive fibres to the total tissue area and calculated using Image-Pro Plus 6.0 (Media Cybernetics Inc., Bethesda, Massachusetts, USA).

### Statistical analysis

Statistical analyses were conducted using SPSS Statistics version 26.0 (IBM Corp., Armonk, NY, USA) and GraphPad Prism 8.0.1 software. All continuous variables are expressed as mean ± standard deviation (SD) or median (interquartile [IQR]), depending on whether these data conform to a normal distribution, as assessed by the Shapiro–Wilk test. Categorical variables are presented as frequencies. Continuous variables conforming to a normal distribution between two groups were compared using the Student’s t-test, while continuous variables conforming to a normal distribution among three or more groups were compared using a one-way analysis of variance (ANOVA). For continuous variables that did not conform to a normal distribution, a nonparametric rank sum test was performed. Pearson’s X^2^ or Fisher’s exact test was used to compare differences in categorical variables. Correlations between tissue stiffness and dysmenorrhea and between the extent of fibrosis and the level of TGF-β, α-SMA, and PGP9.5 were assessed by Pearson’s coefficient. *P*-values < 0.05 were considered statistically significant.

## Results

**Fig. 1:**
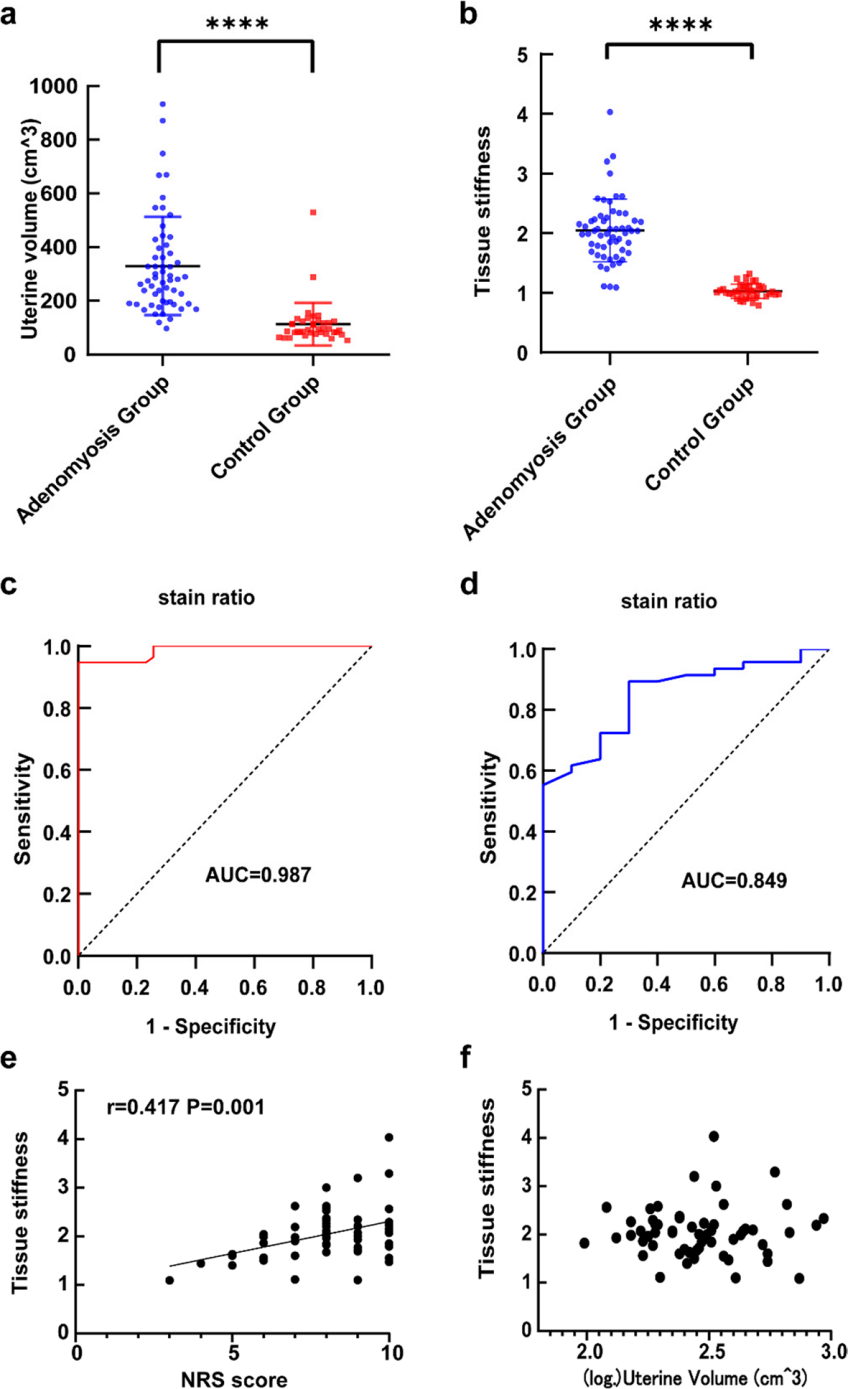
**a** The uterine volume in adenomyosis group and control group; **b** The tissue stiffness assessed by elastography of adenomyosis lesion in adenomyosis group and normal myometrium in control group; **c** The receiver operating characteristic(ROC)curve analysis to evaluate the cutoff value of tissue stiffness for adenomyosis; **d** ROC curve analysis to evaluate the cutoff value of tissue stiffness for heavy dysmenorrhea; **e** Correlation between NRS score and lesion tissue stiffness in adenomyosis group; **f** Correlation of uterine volume and lesion stiffness in adenomyosis group. The results represent the mean ± SD. **** P < 0.0001

The demographic and clinical characteristics of the patients are listed in Table 1. Ninety-six patients were enrolled in this study: 57 with adenomyosis (adenomyosis group) and 39 without myometrial lesions (control group). Patients with adenomyosis had higher dysmenorrhea scores than those of the control group. The uterine volume was significantly different between the adenomyosis and control groups (280.22 and 91.27 cm^3^, respectively) (Fig. 1a), whereas no differences were found in age (*P* = 0.217) or BMI (*P* = 0.122). Patients with adenomyosis mainly presented with severe dysmenorrhea (82.46%) and heavy menstrual bleeding (75.44%), whereas patients in the control group mainly had none or mild dysmenorrhea (97.44%) and moderate menstruation (64.10%).

**Table 1:** Characteristics of recruited patients in the study

	Adenomyosis Group	Control Group	*P* Value
Case number, n	57	39	
Age, years	41.30 ± 4.86	39.63 ± 6.29	0.217
BMI, kg/m^2^	24.80(21.67–28.19)	24.47(21.37–25.34)	0.122
Dysmenorrhea			< 0.001
None/Mild	1	38	
Moderate	9	1	
Severe	47	0	
NRS score for dysmenorrhea	7(8–9)	0	< 0.001
Amount of menses			
Light	3	8	< 0.001
Moderate	11	25	
Heavy	43	6	
Uterine size, cm^3^	280.22(192.05–402.39)	91.27(80.43–119.61)	< 0.001
stain ratio	2.04(1.71–2.25)	1.02(0.96–1.09)	< 0.001

### Higher stiffness of adenomyosis lesions than control normal myometrium

**Fig. 2:**
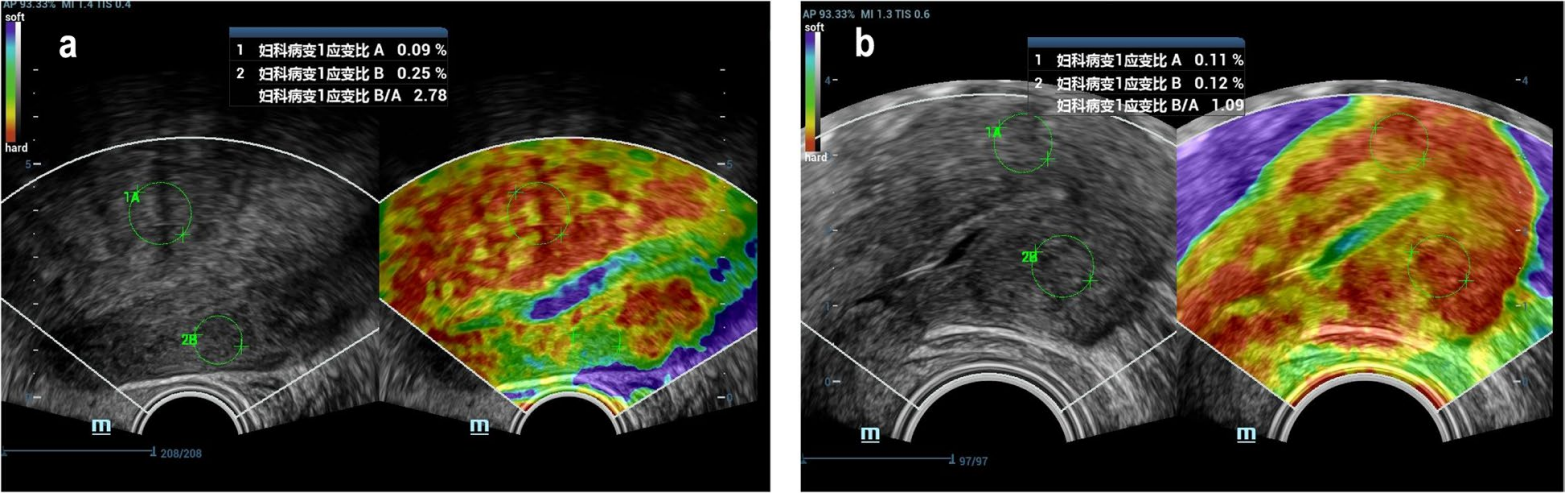
Elastosonographic image of adenomyosis and normal myometrium. **a** elastosonographic image of adenomyosis; **b** elastosonographic image of normal myometrium. The adenomyotic lesion is mainly red-coded and stiffer than the adjacent normal myometrium with indefinite boundary. Normal myometrium showed uniform color-coded in elastosonographic image. Note: “妇科病变1应变比A” meant the stain percentage of gynecological lesion 1 (adenomyotic lesion), “妇科病变2应变比” meant the stain percentage of gynecological lesion 1(the adjacent normal myometrium). “妇科病变1应变比B/A” represented the stifness of adenomyotic lesion calculated by the ratio of stain percentage between the lesion and the adjacent normal myometrium, which is mainly coded by green

An enlarged spherical uterus with inhomogeneous echogenicity within the lesion or focal inhomogeneous echogenicity within the myometrium with no clear boundary to the adjacent normal myometrium was observed in the TVUS images of the adenomyosis group (Fig. 2a). Elastograms of adenomyotic lesions were encoded mainly in red, while those surrounding normal myometrium in adenomyosis patients were encoded in very green and slightly yellow colours. The myometrial echogenicity in the control group was homogeneous, and elastograms of the myometrium were encoded mainly in red and yellow (Fig. 2b). The stiffness, calculated as Ref/ROI (or ROI1/ROI2), was significantly higher in adenomyotic lesions than in the normal control myometrium (Fig. 1b). The cutoff for the strain ratio between the adenomyosis and control groups was > 1.36, with a sensitivity of 94.7%, specificity of 100%, and area under the curve (AUC) of 0.987 (95% confidence interval [CI], 0.97–1.00). Also, the cutoff for the strain ratio between adenomyosis patients with mild-to-moderate dysmenorrhea and those with severe dysmenorrhea was > 1.65; the sensitivity and specificity were 90.0% and 70.0%, respectively, and the AUC was 0.849 (95% CI, 0.74–0.96) (Fig. 1c–d). In the adenomyosis group, the NRS score, representing the degree of dysmenorrhea, correlated positively with the lesion stiffness (Fig. 1e), whereas no correlation was observed between the NRS score and the uterine or lesion volume. There was also no significant correlation among lesion stiffness, uterine volume, and lesion volume (Fig. 1f and Additional file 1).

### The extent of fibrosis in adenomyosis and its correlation with TGF-β and α-SMA

**Fig. 3:**
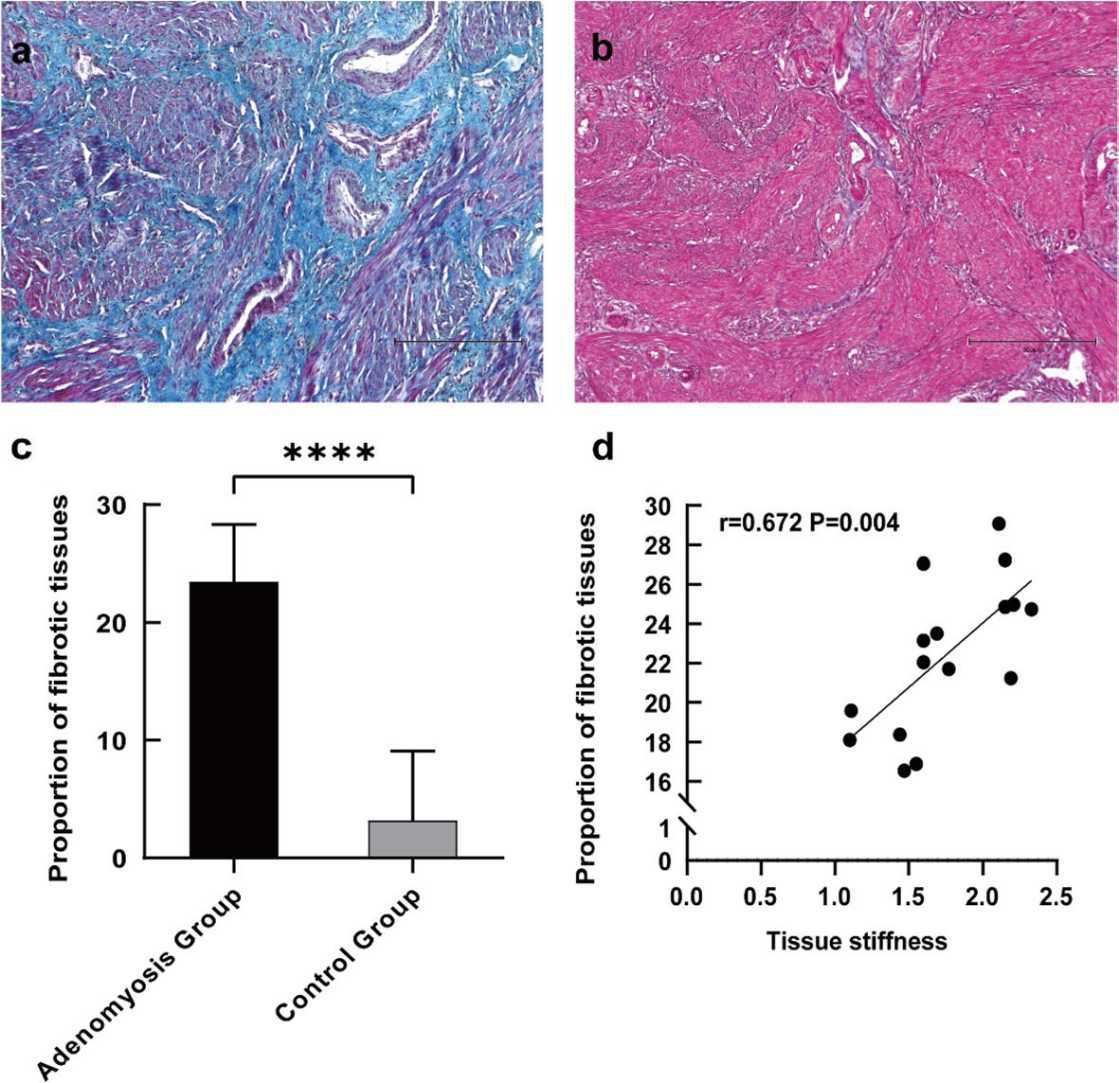
**a** Representative micrographs (100 ×) of collagen fiber staining in the adenomyosis lesion of adenomyosis group; **b** Representative micrographs (100 ×) of collagen fiber staining in the normal myometrium of control group; **c** quantitative analysis of the mean area ratio of collagen fiber between the two groups; **d** Correlation between tissue stiffness and the mean area ratio of collagen fiber. The results represent the mean ± SD. **** P < 0.0001

**Fig. 4:**
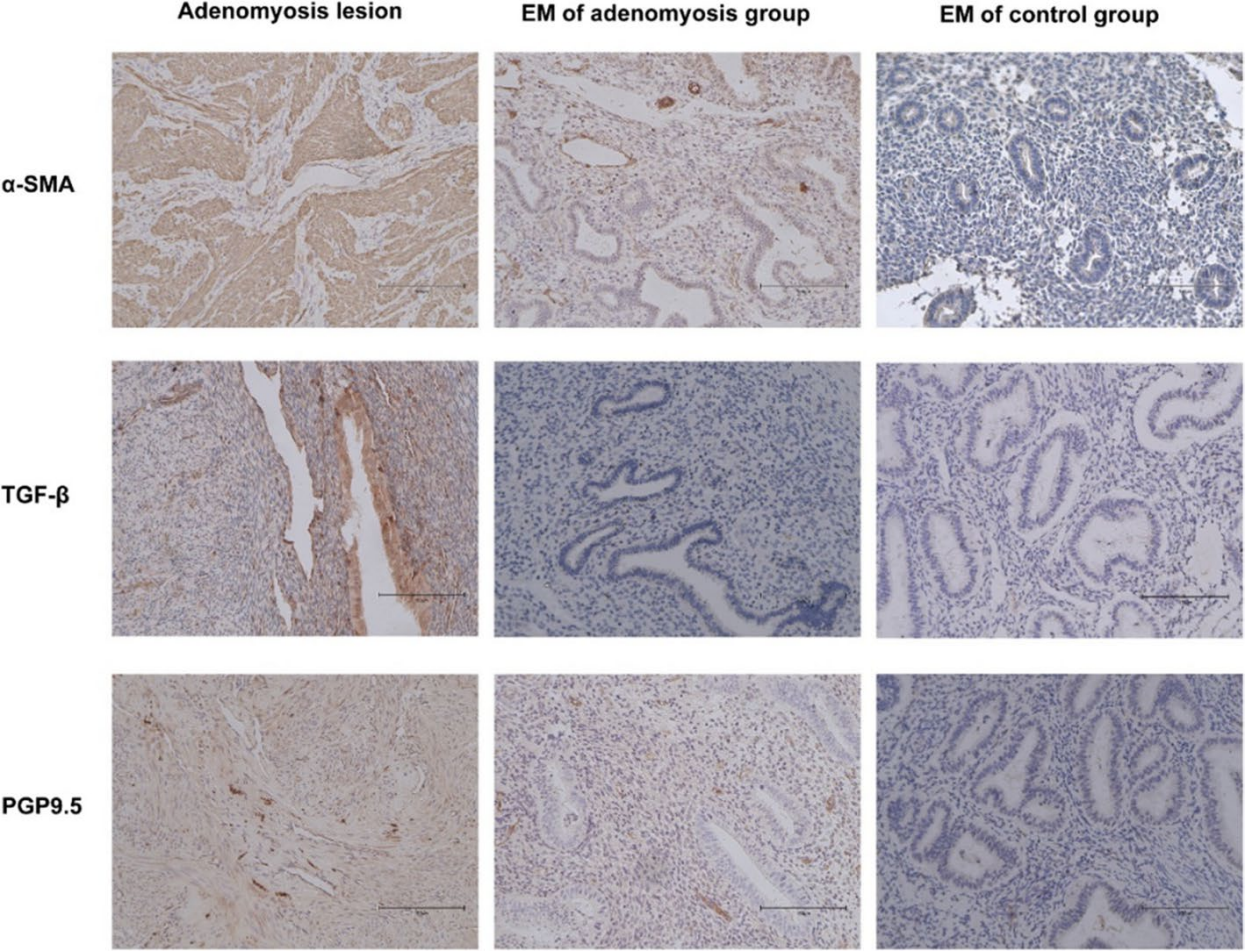
Representative micrographs (200 ×) of α-SMA, TGF-β, PGP9.5 immunostaining in the adenomyosis lesion, EM of patients with adenomyosis and EM of control subjects. EM = endometrium

**Fig. 5:**
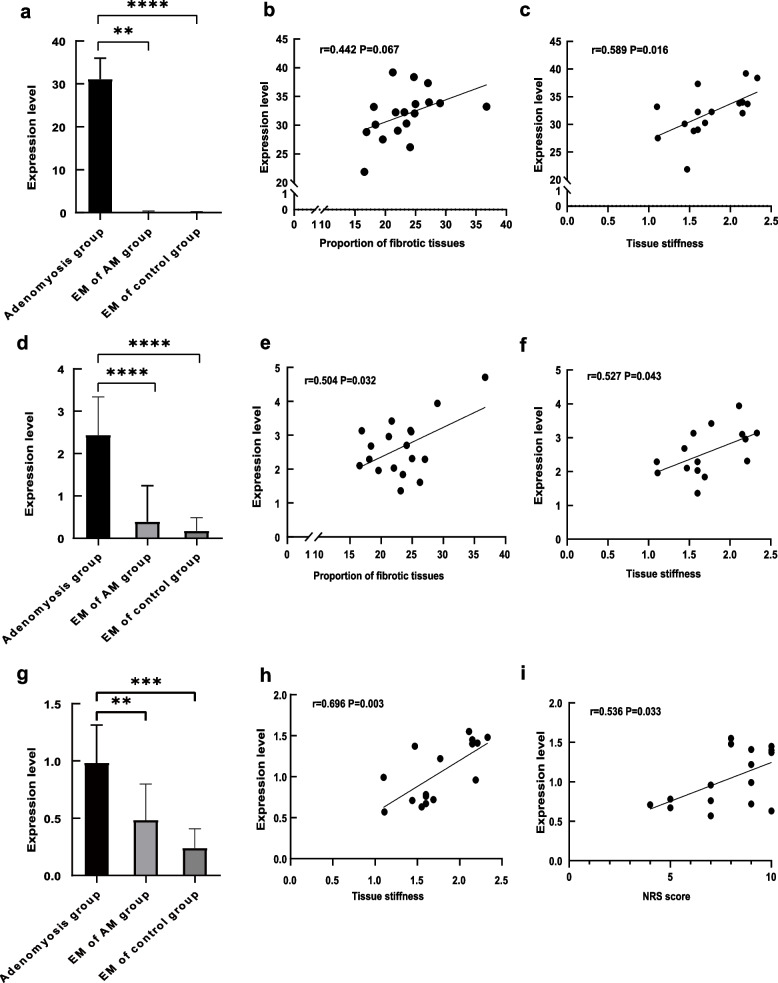
**a** Expression level of α-SMA in adenomyosis lesion, EM of patients with adenomyosis and EM of control group; **b** Correlation between proportion of fibrotic tissue and expression level of α-SMA; **c** Correlation between tissue stiffness and expression level of α-SMA; **d** Expression level of TGF-β in adenomyosis lesion, EM of patients with adenomyosis and EM of control group; **e** Correlation between proportion of fibrotic tissue and expression level of TGF-β; **f** Correlation between tissue stiffness and expression level of TGF-β; **g** Expression level of PGP9.5 in adenomyosis lesion, EM of patients with adenomyosis and EM of control group; **h** Correlation of tissue stiffness with expression level of PGP9.5; **i** Correlation between NRS score and expression level of PGP9.5. The results represent the mean ± SD. **P < 0.01 ***P < 0.001 ****P < 0.0001. EM = endometrium, AM = adenomyosis

We further evaluated the correlation of tissue stiffness with the extent of fibrosis and the expression of TGF-β and α-SMA. The extent of fibrosis was significantly higher in adenomyotic lesions than in normal myometrium in the control group (Fig. 3a-c). In addition, the extent of fibrosis showed a significant positive correlation with the tissue stiffness of the adenomyotic lesions (Fig. 3d). TGF-β staining was mostly seen in the cytoplasm of glandular epithelial cells, with some in the stromal cells of adenomyotic lesions, while little or no staining was observed in control endometrium. Similarly, α-SMA staining was also primarily observed in the cytoplasm of stromal cells of adenomyotic lesions, with no staining in the control endometrium (Fig. 4). Expression levels of both TGF-β and α-SMA were significantly higher in adenomyotic lesions compared with those in the eutopic endometrium of both the adenomyosis and control groups (Fig. 5a, d). Further, a positive correlation trend was found between the expression level of α-SMA and the extent of fibrosis in adenomyotic lesions, although no statistical difference was observed (Fig. 5b). The expression level of TGF-β showed a significantly positive correlation with the extent of fibrosis in adenomyotic lesions (Fig. 5e). Both TGF-β and α-SMA expression levels were significantly positively correlated with tissue stiffness (Figs. 5c, f).

### Higher expression level of PGP9.5 in adenomyosis

The expression level of PGP9.5 was also evaluated to assess the degree of dysmenorrhea. PGP9.5 staining was majorly observed in the cytoplasm of adenomyotic lesions and eutopic endometrium of the adenomyosis group compared with no staining in the control endometrium (Fig. 4). Expression levels in the adenomyotic lesions were higher than those in the eutopic endometrium of the adenomyosis group, both of which were higher than those in the control endometrium (Fig. 5g). The expression level of PGP9.5 in the adenomyosis group was significantly positively correlated with the degree of dysmenorrhea, as evaluated by the NRS score (Fig. 5i). Similarly, a positive correlation between the expression level of PGP9.5 and tissue stiffness was also found (Fig. 5h).

## Discussion

ReTIAR plays an important role in the development of adenomyosis [4–6]. According to ReTIAR, continuous tissue damage and repair lead to EMT, FMT, and finally, fibrosis, thus leading to the development and exacerbation of adenomyosis. Elastography, an imaging tool to evaluate tissue stiffness, can be used to measure lesion stiffness and assess the progression of adenomyosis [6, 15, 16]. Elastography has been shown to improve the sensitivity and specificity of the ultrasound diagnosis of adenomyosis and has great value in the differential diagnosis of adenomyosis and uterine fibroids [6, 16–18]. The stiffness of adenomyotic lesions is higher than that of uterine fibroids, both of which are higher than that of normal myometrium; these results are consistent with our findings. In addition, we found that the lesion stiffness was positively correlated with the degree of dysmenorrhea in patients with adenomyosis, with a higher lesion stiffness in patients with severe disease. This result indicates that the degree of dysmenorrhea in patients with adenomyosis can be objectively evaluated by tissue stiffness measured using elastography instead of subjective scoring tools such as NRS score and Visual Analogue Scale score, which better evaluate the severity of dysmenorrhea in patients with adenomyosis.

Although Guo et al. [6] found a positive correlation between lesion stiffness and uterine volume, our study found no significant correlation between either the degree of dysmenorrhea or lesion stiffness and the uterine or lesion volume. These results explain the fact that some patients have a large uterus but no obvious symptoms, while others show significant symptoms such as dysmenorrhea and/or menorrhagia without a large uterus, suggesting that it may be unreasonable to evaluate the degree of adenomyosis solely based on the uterine or lesion volume.

Currently, no criteria for evaluating the severity or staging of adenomyosis are available. Lazzeri et al. designed an ultrasound mapping system to evaluate the severity of adenomyosis based on the type of adenomyosis, the size of the lesion, and the involvement of the junctional zone [19, 20]. Although the classification system was relatively comprehensive and showed good interobserver agreement, it did not incorporate the elastography findings and may not be convenient for clinical application. Our results and those of some previous studies have demonstrated the value of elastography in diagnosing and assessing the severity of endometriosis and adenomyosis [6, 11, 21–23]. Elastography is also valuable in evaluating the efficacy of conservative treatment. Chiara et al. [24] studied the value of real-time elastography using a transvaginal approach and assessed the response of uterine fibroids to magnetic resonance-guided focused ultrasound surgery treatment. They found a reduction in the strain ratio (ROI lesions/ROI normal myometrium) for fibroids after treatment compared to that before treatment.

PGP9.5 is widely expressed at all stages of neuronal differentiation and is an important and highly specific marker that is widely used to label nerve fibres [25, 26]. PGP9.5 also plays a role in neural regeneration and the regulation of tumour cell invasion [27, 28]. In this study, we found that PGP9.5 was highly expressed in the eutopic and ectopic endometrium of patients with adenomyosis. Thus, PGP9.5 may play an important role in the metastasis and invasion of the eutopic endometrium and the continued growth and infiltration of the ectopic endometrium, leading to the progression of adenomyosis and a worsening of dysmenorrhea.

TGF-β, as a typical pro-fibrogenic cytokine, plays a crucial role in organ fibrosis, including the progression of adenomyosis [29, 30]. Various exogenous and endogenous factors cause the uterus to undergo ReTIAR [31, 32], which leads to the release of a number of bioactive factors, such as TGF-β. Activated TGF-β promotes the progression of FMT through the TGF-β1/Smad signalling pathway, ultimately leading to the regulation of α-SMA expression and increased deposition of extracellular matrix components, such as collagen. Meanwhile, increased fibrosis leads to myometrial tissue stiffening, which affects myometrial contraction and causes dysmenorrhea. TGF-β can also induce the expression of PGP9.5 [27], enhancing the ability of the eutopic endometrium to invade the myometrium and worsening the degree of dysmenorrhea. Akishima-Fukasawa et al. also found that the expression of both PGP9.5 and α-SMA was increased by TGF-β stimulation and blocked by neutralization of TGF-β with anti-TGF-β antibody [33]. Additionally, the study found that PGP9.5 + fibroblasts occur primarily in dense fibrotic regions with less cancer cell invasion or in fibrotic regions in stroma with abundant extracellular matrix. Our results also showed that PGP9.5 expression was positively correlated with tissue stiffness. These results indicate that various exogenous and endogenous factors stimulate the uterine release of various bioactive factors, including TGF-β. On the one hand, TGF-β promotes fibrosis [32], further influencing the uterine contraction ability and leading to dysmenorrhea. On the other hand, TGF-β can induce the expression of PGP9.5, resulting in nerve fibre invasion into the myometrium and, finally dysmenorrhea.

However, this study had some limitations. First, the number of participants was relatively small; therefore, validation in a larger population is required. Secondly, both Masson’s trichrome staining and IHC are semi-quantitative analysis techniques, which cannot definitely assess the level of fibrosis and expression levels of TGF-β, α-SMA, and PGP9.5.

## Conclusion

Our results showed that elastography has good specificity and sensitivity to the diagnosis of adenomyosis and heavy dysmenorrhea in patients with adenomyosis. Elastography can be used to assess the stiffness of adenomyotic lesions, which is positively correlated with the severity of dysmenorrhea. Furthermore, we found that the degree of dysmenorrhea was not related to the uterine or lesion volume, which indicates that using the uterine or lesion volume alone to assess the severity of adenomyosis is inappropriate. Further studies should examine the value of elastography in assessing the efficacy of conservative treatments for adenomyosis, such as dienogest, gonadotropin-releasing hormone agonists and, et al.

### Supplementary Information

**Additional file 1.**

## Data Availability

The datasets supporting the conclusions of this article are available from the authors on reasonable request.

## Abbreviations

α-SMA: α-Smooth muscle actin
ANOVA: Analysis of variance
AUC: Area under the curve
BMI: Body mass index
CI: Confidence interval
EMT: Epithelial-mesenchymal transition
FMT: Fibroblast-to-myofibroblast transition
H&E: Hematoxylin and eosin
HRP: Horseradish peroxidase
IHC: Immunohistochemistry
IQR: Interquartile range
MRI: Magnetic resonance imaging
NRS: Numerical rating scale
PGP9.5: Protein gene product 9.5
ReTIAR: Repeated tissue injury and repair
ROI: Region of interest
SD: Standard deviation
TGF-β: Transforming growth factor-β
TVUS: Transvaginal ultrasound
