# Supplementary material for: Application of elastography to diagnose adenomyosis and evaluate the degree of dysmenorrhea: a prospective observational study
Source: Reprod Biol Endocrinol. 2023 Oct 26;21:98. doi: 10.1186/s12958-023-01145-y (PMC10601167; doi:10.1186/s12958-023-01145-y)
Supplement: Supplementary file 1 — Additional file 1. [file 12958_2023_1145_MOESM1_ESM.docx]

Supplementary information


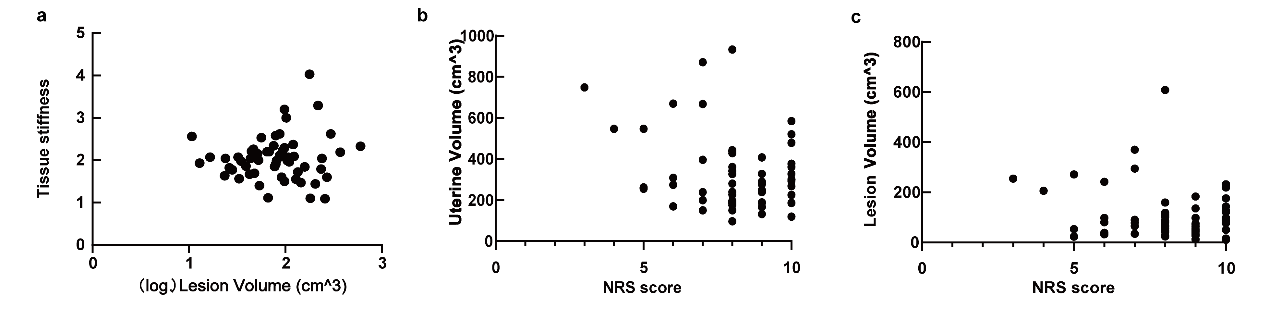


a. Correlation of lesion volume and lesion stiffness in adenomyosis group; b. Correlation of NRS score and uterine volume in adenomyosis group; c. Correlation between NRS score and lesion volume in adenomyosis group.
